# Targeting Ferroptosis as the Achilles’ Heel of Breast Cancer: Mechanisms and Therapeutic Opportunities from a Comprehensive Systematic Review

**DOI:** 10.3390/ijms26209902

**Published:** 2025-10-11

**Authors:** Anna Szulc, Marta Woźniak

**Affiliations:** Department of Clinical and Experimental Pathology, Division of General and Experimental Pathology, Wroclaw Medical University, 50-368 Wroclaw, Poland; anna.szulc@umw.edu.pl

**Keywords:** ferroptosis, breast cancer, iron metabolism, lipid peroxidation, tumor microenvironment, triple-negative breast cancer, chemoresistance, metabolic reprogramming, therapeutic targeting

## Abstract

Ferroptosis, an iron-dependent form of regulated cell death marked by lipid peroxidation, has emerged as a promising therapeutic target in breast cancer, particularly in aggressive subtypes such as triple-negative breast cancer (TNBC). This systematic review explores the molecular mechanisms underlying ferroptosis sensitivity and resistance, focusing on the interplay between iron metabolism, antioxidant defenses, and tumor microenvironmental factors. Literature retrieved from PubMed and Scopus up to May was analyzed in accordance with PRISMA guidelines, including mechanistic studies, preclinical experiments, and ongoing clinical trials. Findings reveal that breast cancer cells evade ferroptosis through enhanced glutathione synthesis, upregulation of GPX4 and system Xc- and adaptive metabolic reprogramming; yet these same mechanisms create exploitable vulnerabilities, including dependence on cystine, polyunsaturated lipids, and dysregulated iron handling. Therapeutic strategies that target key ferroptosis regulators, such as GPX4, ACSL4, and SLC7A11, or that harness agents like statins, sulfasalazine, and nanoparticle-based iron complexes demonstrate strong potential to overcome chemoresistance and selectively eliminate therapy-resistant cancer cell populations. Taken together, the evidence highlights ferroptosis as a critical Achilles’ heel of breast cancer biology and supports further clinical translation of ferroptosis-inducing therapies to improve outcomes in otherwise refractory breast cancer subtypes.

## 1. Introduction

Breast cancer remains a significant global health issue, being the most frequently diagnosed cancer among women. In 2022, the World Health Organization (WHO) reported approximately 2.3 million new cases and over 670,000 deaths [1]. Although various treatment modalities—including surgical resection, radiotherapy, chemotherapy, endocrine therapy, and targeted therapy—are utilized, patient outcomes are still suboptimal [2]. Despite considerable advancements in breast cancer therapies over the past few decades, the management of metastasis and recurrence continues to pose the greatest challenge [3]. Clinically, breast tumors are categorized based on the expression of estrogen receptor (ER), progesterone receptor (PR), and human epidermal growth factor receptor 2 (HER2/ERBB2) into three main subtypes: ER-positive, HER2-positive, and triple-negative breast cancer (TNBC) [4]. The latter is recognized as the most aggressive subtype of breast cancer, characterized by rapid disease onset, high rates of recurrence after treatment, and an abysmal prognosis [5]. Due to its distinct cellular phenotype, TNBC lacks key receptors and targets, making it unresponsive to HER2-targeted therapies and hormonal treatments [6]. As a result, chemotherapy remains the primary therapeutic approach for TNBC [7]. However, the majority of TNBC patients eventually develop chemoresistance, either through intrinsic or acquired resistance to apoptosis, leading to treatment failure, recurrence, and accelerated disease progression [8]. Chemoresistant cells, selected during treatment, contribute significantly to therapeutic failure, distant metastasis, and tumor relapse, with factors such as metabolic reprogramming following chemotherapy playing a critical role in this selection process [9].

In addition to the fundamental aggressiveness of breast cancer subtypes like TNBC, other factors, such as breast density, significantly complicate early detection and prognosis. Dense breast tissue is one of the strongest independent risk factors for breast cancer, apart from age and genetics [10]. Women with extremely dense breasts face a four- to sixfold higher risk of developing breast cancer compared to those with predominantly fatty breasts [11]. Moreover, dense breast tissue significantly increases the likelihood of interval cancers, which are tumors that develop between routine mammograms and are 17.8 times more likely to occur in dense breasts [12]. As breast density increases, mammography sensitivity declines sharply—from 93% in fatty breasts to as low as 30% in extremely dense ones, making it harder to identify non-calcified cancers early [13]. Because both dense tissue and tumors appear similarly on mammograms, cancer can be obscured, leading to underdiagnosis or late detection [14]. This delay, particularly in women with dense breast tissue, can not only result in larger tumors but also contribute to the formation of necrosis. Under conditions of severe nutrient deprivation and compromised vasculature, tumor cells undergo necrotic cell death [15]. Necrosis is a common characteristic of highly aggressive, rapidly proliferating breast tumors and is linked to a poor prognosis as well as a significantly elevated risk of metastasis [16,17]. Necrosis generally becomes evident once a solid tumor exceeds 4 mm in diameter. The necrotic regions within the tumor core are challenging to target with conventional treatments like radiation or chemotherapy due to their resistance to these modalities [18]. Consequently, there is an urgent need to understand the mechanisms underlying metabolic reprogramming better and to develop effective non-apoptotic therapeutic strategies, such as targeting ferroptosis, for the treatment of breast cancer.

Cell death is critical for maintaining the balance of biological processes, ensuring normal development and tissue homeostasis, and preventing uncontrolled cell growth, such as in cancer. Programmed cell death regulates tissue renewal and immunological responses, which is fundamental in preventing illnesses caused by excessive cell growth [6]. Apoptosis is the most extensively studied form of regulated cell death, primarily initiated through the activation of enzymes known as caspases [19]. Recently, research has increasingly focused on non-apoptotic forms of cell death, driven by the recognition that evading apoptosis is a hallmark of cancer [20]. In 2012, a significant advancement in cell death research occurred with introducing the term “ferroptosis.” This discovery marked a pivotal moment, as ferroptosis was recognized as a novel, non-apoptotic form of regulated cell death driven by iron-dependent lipid peroxidation [21]. Three key factors determine the sensitivity of cells to ferroptosis: the presence of oxidizable phospholipids (PLs) that are enriched with polyunsaturated fatty acids (PUFA-PLs), the availability of redox-active iron, and impairments in lipid peroxide repair mechanisms. The concurrent presence of all three factors is essential for ferroptosis to occur, as each plays a critical role in driving the lipid peroxidation and cellular damage characteristic of this form of cell death [22].

Yet another ferroptosis feature is the distinct mitochondrial alterations such as decreased mitochondrial volume, increased membrane density, outer membrane rupture, and the disappearance of cristae [23]. These changes signal mitochondrial involvement in ferroptotic progression, which is linked to the generation of reactive oxygen species (ROS) within the organelle [24]. Since mitochondria are central to cellular metabolism and oxidative phosphorylation, they are also key contributors to ROS production—critical in triggering lipid peroxidation. To better understand why mitochondrial respiration becomes rampant, the biochemical control of ferroptosis needs to be discussed. The primary enzyme that regulates lipid peroxidation is glutathione peroxidase 4 (GPX4), which catalyzes the reduction of harmful phospholipid hydroperoxides into non-toxic phospholipid alcohols [25]. This process relies on glutathione (GSH), a tripeptide composed of cysteine, glutamate, and glycine, the most abundant antioxidant within cells. The system Xc–, an amino acid transporter made up of SLC7A11 and SLC3A2 subunits, plays a crucial role in this process by importing cystine into the cell and exporting glutamate. Once inside the cell, cystine is rapidly reduced to cysteine, the rate-limiting precursor for GSH synthesis, ensuring the availability of GSH for lipid peroxide detoxification [26]. Accordingly, inhibiting system Xc- or depriving cells of cystine results in a depletion of GSH, which in turn diminishes the cell’s ability to neutralize reactive oxygen species (ROS) [27,28]. Consequently, oxidative stress increases, and glutamate, which is no longer being exported, accumulates within the cell. Excess intracellular glutamate is converted into α-ketoglutaric acid (α-KG), a key intermediate in the tricarboxylic acid (TCA) cycle. This increase in α-KG drives the TCA cycle more intensely, resulting in heightened mitochondrial activity. The hyperactivation of the TCA cycle leads to the hyperpolarization of the mitochondrial membrane potential, thereby accelerating mitochondrial respiration and contributing to the rampant oxidative damage associated with ferroptosis [29].

A distinctive characteristic of ferroptosis is the absence of involvement of death receptors or sensors in initiating the ferroptotic pathway [30]. Instead, ferroptosis is triggered when the cell’s antioxidant defense systems are unable to prevent the oxidative degradation of lipid membranes, resulting in membrane damage, rupture, and eventual necrotic cell death [30]. Increasing evidence has linked disruptions in ferroptotic pathways to diseases associated with iron overload or reactive oxygen species (ROS), including cancer, neurodegenerative conditions, infections, and inflammatory disorders [31,32,33]. The objective of this study is to explore the intricate role of ferroptosis in breast cancer, with a particular focus on the tumor microenvironment and iron metabolism. These elements present a paradox in breast cancer, acting both as facilitators of tumor growth—by meeting the heightened metabolic demands of rapidly proliferating cells—and as critical players in the cancer cells’ defense against ferroptosis due to their ability to undergo metabolic reprogramming. At the same time, ferroptosis represents a potential vulnerability, or “Achilles’ heel,” for breast cancer, offering a novel therapeutic avenue. Furthermore, we will present an up-to-date review of ongoing clinical trials targeting ferroptosis in breast cancer, highlighting the therapeutic potential of manipulating ferroptotic pathways in this context.

**Figure 1:**
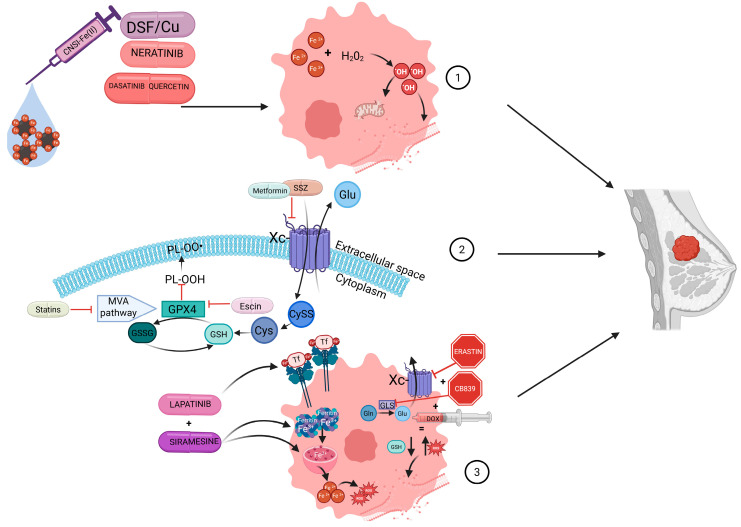
Strategies for Inducing Ferroptosis in Breast Cancer Cells through Iron Metabolism and Antioxidant Disruption. 1. Therapeutic agents such as DSF/Cu, neratinib, dasatinib, quercetin, and CNSI-Fe(II) promote intracellular Fe^2+^ accumulation and enhance reactive oxygen species (ROS) production via the Fenton reaction, leading to ferroptotic damage in breast cancer cells. 2. Inhibition of the antioxidant defense system through blockade of the system Xc- transporter (e.g., sulfasalazine, metformin) prevents cystine (CySS) uptake, reducing glutathione (GSH) synthesis. Additionally, GPX4 inhibitors (e.g., statins, escin) or upstream MVA pathway blockade compromise lipid peroxide detoxification, enhancing ferroptotic susceptibility. 3. Targeting iron uptake and GSH metabolism, agents like erastin, CB839, and doxorubicin impair glutaminolysis and glutathione synthesis. Concurrently, lapatinib and siramesine promote lysosomal ferritin degradation (ferritinophagy), increasing the labile iron pool and ferroptotic stress.

In Figure 1 we present the graphical abstract.

## 2. Materials and Methods

**Figure 2:**
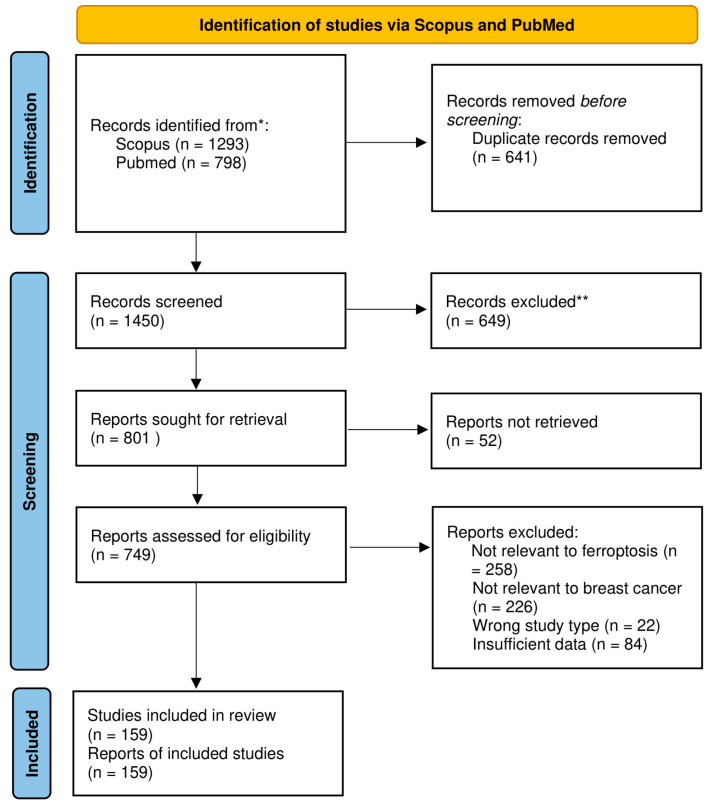
PRISMA 2020 flow diagram. * Consider, if feasible to do so, reporting the number of records identified from each database or register searched (rather than the total number across all databases/registers). ** If automation tools were used, indicate how many records were excluded by a human and how many were excluded by automation tools.

This review was prepared in accordance with PRISMA (Preferred Reporting Items for Systematic Reviews and Meta-Analyses) guidelines. A comprehensive literature search was performed until May using the PubMed and Scopus databases. The search terms “ferroptosis” and “breast cancer” were applied to titles, abstracts, and keywords to identify relevant studies. Only articles written in English were considered. Studies discussing the role of ferroptosis in breast cancer—including mechanistic research, therapeutic strategies, and relevant in vivo and in vitro studies—were included. In addition to database searches, selected clinical trial reports were identified manually. This review was not registered in PROSPERO or any other registry. Review protocol was not prepared. Formal risk of bias assessment was not conducted, as the included studies were highly heterogeneous in design and outcomes, and the review was intended as a narrative synthesis. Nevertheless, potential sources of bias across the included studies are addressed narratively in Section 3.4. No quantitative effect measures were used because no meta-analysis was performed; findings were synthesized narratively. Data were summarized narratively and supported with tables and figures. No subgroup, heterogeneity, or sensitivity analyses were conducted, given the descriptive scope of this review. The process of study selection is detailed in Figure 2.

## 3. Results and Discussion

### 3.1. Tumor Microenvironment

Cancer cells, unlike their normal counterparts, undergo significant metabolic reprogramming to support the rapid proliferation characteristic of tumor growth [34]. One of the primary distinctions between cancer cells and healthy cells is their glucose metabolism. Healthy cells preferentially utilize oxidative phosphorylation under aerobic conditions, as it is a highly efficient method for ATP production. In contrast, cancer cells often exist in a hypoxic environment, which drives alterations in mitochondrial function, enabling them to generate energy primarily through aerobic glycolysis—a process known as the Warburg effect, first identified by Otto Warburg in the 1920s [35,36]. This shift in metabolism has profound implications for the tumor microenvironment, as the rapid cell growth coupled with high lactate production can outpace the available blood supply and lead to acidification of the surrounding tissues [37]. As a compensatory advantage, the altered glucose metabolism in cancer cells not only meets their energy demands but also provides essential intermediates for biosynthetic pathways. These include ribose sugars for nucleotide synthesis, glycerol and citrate for lipid production, and nonessential amino acids, all of which are crucial for supporting the rapid growth and proliferation of tumor cells [38]. Lactate, a by-product of glycolysis, builds up in the tumor microenvironment (TME), creating an acidic milieu that hinders antitumor immune responses. This suppression occurs through the dysfunction of cytotoxic T cells and natural killer (NK) cells, decreased cytokine production, and the promotion of immunosuppressive cell populations, including regulatory T cells (Tregs) and tumor-associated macrophages (TAMs). Lactate’s immunosuppressive influence is further intensified by its contribution to angiogenesis, epithelial-to-mesenchymal transition (EMT), and extracellular matrix remodeling. Moreover, lactate-driven metabolic reprogramming aids tumor survival in hypoxic conditions [39]. These processes highlight how metabolic adaptations, such as lactate accumulation, shape the TME and support cancer progression.

Breast cancer, a highly heterogeneous disease at both the molecular and clinical levels, exemplifies how metabolic and oxidative stress influence cancer progression. Cancer cells must navigate this altered environment, where the balance between metabolic stress and antioxidant defenses determines whether the conditions will inhibit or promote their growth [40]. Depending on the severity of stress and the tumor’s internal capacity for managing oxidative damage, these changes may either hinder or facilitate cancer cell survival and proliferation [41]. Thus, the metabolic adaptations of cancer cells, including the Warburg effect, play a critical role in shaping the microenvironment that supports tumor development and progression.

In the TME of breast cancer, adipocytes have garnered significant interest due to their close association with developing tumors [42]. Breast cancer cells interact with mammary adipocytes, creating a distinct microenvironment that supports tumor survival [43]. Adipocytes influence lipid metabolism in breast cancer cells, with alterations in lipid remodeling observed. The release of free fatty acids (FFA) and the presence of lipid droplets have been implicated in enhancing the invasive potential of breast cancer cells [44]. Adipocytes contribute to the growth of cancer cells through two primary mechanisms. First, they secrete adipocytokines—growth factors and cytokines that activate signaling pathways in tumor cells, promoting various oncogenic processes. Notably, interleukin-6 (IL-6) and leptin released by adipocytes regulate epithelial–mesenchymal transition (EMT) in cancer cells, while leptin also enhances stem cell renewal and increases drug resistance [45,46,47]. Second, adipocytes release metabolites and biomolecules that alter cancer cell metabolism to promote tumor growth. Adipocytes that are in direct contact with tumor cells, referred to as cancer-associated adipocytes (CAAs), exhibit distinct characteristics compared to normal adipocytes [48]. For instance, in breast cancer, adipose tissue infiltrated by tumors shows a significant reduction in lipid droplet (LD) size, while CAAs demonstrate elevated expression of IL-6 and leptin [49,50]. The close interaction between CAAs and cancer cells allows breast cancer cells to utilize metabolites such as lactate, glutamine, and fatty acids (FAs) from the adipocytes, providing an energy source and metabolic advantage in the nutrient-restricted tumor microenvironment [38,51,52]. These metabolites support cancer cell survival and progression, particularly in challenging conditions.

In addition to metabolic reprogramming, apoptosis and autophagy represent fundamental processes that shape the tumor microenvironment (TME) in breast cancer. Apoptosis is often dysregulated in breast tumors: for example, elevated expression of anti-apoptotic proteins like BCL-2 is a common feature, correlating with more aggressive disease in triple-negative breast cancer (TNBC) and poorer overall survival [53]. BH3 mimetics (which inhibit BCL-2) have been shown to sensitize breast cancer cells to chemotherapies (e.g., docetaxel), by promoting dissociation of pro-apoptotic factors like BIM from BCL-2 and thereby activating caspase-mediated apoptosis [54]. In primary vs. metastatic breast carcinomas, higher apoptotic index tends to associate with more poorly differentiated, larger tumors, higher Ki-67, and other markers of aggressive phenotype, while BCL-2 expression is often more abundant in smaller, hormone receptor-positive tumors [55]. This evasion is particularly pronounced in hypoxic or nutrient-restricted environments, where apoptotic signaling is suppressed, allowing malignant cells to survive and expand despite adverse conditions [56].

Autophagy, a lysosome-mediated degradation pathway, exerts a dual influence on breast cancer progression. On the one hand, it functions as a survival mechanism, enabling tumor cells to recycle macromolecules and sustain proliferation in nutrient-poor and oxidative microenvironments [57,58]. On the other hand, excessive or dysregulated autophagy may trigger cell death, either directly (autophagic cell death) or indirectly by facilitating ferroptosis through ferritinophagy, the selective degradation of ferritin that increases intracellular labile iron [59]. Experimental work further supports this duality; for example, atorvastatin was shown to induce both apoptosis and autophagy in MCF-7 cells [60], while adiponectin was found to stimulate autophagy followed by apoptosis, thereby suppressing breast tumor growth [61].

Importantly, apoptosis and autophagy do not act in isolation. Their signaling frequently overlaps with ferroptosis, particularly in the oxidative and hypoxic niches of breast tumors. Reactive oxygen species generated within the TME can simultaneously trigger mitochondrial apoptosis, activate protective autophagy, or drive lipid peroxidation characteristic of ferroptosis. This convergence highlights that breast cancer cell fate is rarely dictated by a single death pathway but rather by a complex interplay of mechanisms. Harnessing this crosstalk may therefore offer novel therapeutic strategies, particularly for aggressive subtypes such as triple-negative breast cancer.

### 3.2. Iron Metabolism and Ferroptosis in Breast Cancer

Iron has been recognized as having a dual role in cancer development and progression. First, as a potential tumor initiator, iron participates in the Fenton reaction, which facilitates the generation of reactive oxygen species capable of inducing DNA damage and leading to carcinogenic mutations [62]. Second, iron functions as a critical growth factor, as cancer cells have a heightened requirement for iron [63,64]. This elevated demand often drives modifications in iron acquisition and utilization pathways, contributing to the metabolic reprogramming characteristic of cancer cells. Breast cancer cells enhance iron uptake through increased expression of transferrin receptor 1 (TfR1), a cell surface receptor critical for transferrin-mediated iron delivery [65]. In circulation, transferrin (TF) binds two Fe^3+^ ions to form a complex, which subsequently interacts with TfR1 on the cell surface, facilitating iron entry into cells for metabolic use [66]. Additionally, breast cancer cells expand their cytosolic labile iron pool through an alternative pathway involving lipocalin-2, a protein within the lipocalin family known for binding small hydrophobic ligands [67]. Specifically, lipocalin-2 binds bacterial catecholate-type ferric siderophores, such as ferric-enterobactin, the primary siderophore of enteric bacteria [68]. Once bound, lipocalin-2 can deliver iron into cancer cells via interaction with its high-affinity receptors, LCN2R (also known as 24p3R or SLC22A17) or megalin, on the cell surface [69]. In addition to increasing iron uptake, breast cancer cells limit iron export. Iron efflux is controlled by ferroportin (FPN), a membrane protein regulated by the peptide hormone hepcidin [70]. Secreted primarily by the liver, hepcidin binds to a specific extracellular domain on ferroportin, triggering its internalization and subsequent degradation through the proteasome pathway [71]. In breast cancer cells, hepcidin expression is elevated in response to bone morphogenetic protein (BMP) signaling and inflammatory stimuli, such as IL-6 secreted by fibroblasts, which promotes FPN degradation and effectively blocks iron export [72,73]. Pinnix et al. documented a significant decrease in ferroportin levels in both malignant breast cancer cell lines and breast cancer tissue, correlating with an increase in intracellular labile iron [74]. Tumor cells reprogram iron metabolism to satisfy rapid growth and proliferation requirements. Iron is a cofactor for essential enzymes involved in cellular respiration and metabolic pathways, notably within the citric acid cycle and ribonucleotide reductase. Ribonucleotide reductase catalyzes the critical conversion of ribonucleotides to deoxyribonucleotides, a rate-limiting step in DNA synthesis. Additionally, iron is essential for the biosynthesis of macromolecules, which supports the cellular growth and division that drive tumor expansion [75,76].

#### 3.2.1. Defense Mechanisms of Breast Cancer Against Ferroptosis

The dependency on iron in cancer cells can heighten their susceptibility to iron-mediated necrosis, as an excess of iron fosters the buildup of lipid-reactive oxygen species. Iron contributes to ferroptosis through multiple mechanisms. First, ferrous iron (Fe^2+^) undergoes oxidation to ferric iron (Fe^3+^) via the Fenton reaction with hydrogen peroxide (H_2_O_2_), producing reactive hydroxyl radicals that trigger the peroxidation of phospholipids and the formation of lipid radicals [77]. Additionally, Fe^2+^ aids in the decomposition of phospholipid hydroperoxide into alkoxyl phospholipid radicals, which, by reacting with other PUFAs, drive further lipid peroxidation, ultimately leading to the breakdown of cellular membranes [31]. Furthermore, Fe^3+^ is essential for activating lipoxygenases (LOXs), enzymes that catalyze PUFA oxygenation, thereby advancing the ferroptosis process [78]. While breast cancer cells depend on iron for growth and are vulnerable to iron-catalyzed ferroptosis, they also deploy sophisticated defense mechanisms to mitigate this risk, allowing them to escape iron-induced death. One of these defenses is an increased amount of ferritin, an iron-storage protein that acts as a protective buffer against ferroptosis by sequestering excess iron in a bioavailable form that minimizes toxicity [79]. This raises a critical question: How do breast cancer cells utilize ferritin and other protective strategies to balance their iron needs with survival? Understanding these defenses offers insight into both the resilience and the potential weaknesses of breast cancer cells in the context of ferroptosis. Shpyleva et al. observed a correlation between elevated levels of ferritin heavy chain (FTH1) and ferritin light chain (FTL) and a reduction in the labile iron pool (LIP), particularly in highly aggressive breast cancer cells, such as MDA-MB-231 cells [80]. Furthermore, FTH1 has been detected at substantially increased levels within the chromatin-bound nuclear fraction of these cells [80]. Prior research had demonstrated that FTH1 can translocate into the cell nucleus, where it protects DNA from iron-induced toxicity [81]. These findings indicate that higher ferritin levels may constitute a critical defense mechanism against iron toxicity in cancer cells, fostering a cellular environment supporting progression toward a more aggressive tumor phenotype. Additionally, decreased intracellular iron levels may drive angiogenesis and tumor progression through hypoxia-inducible factor 1-alpha (HIF-1α)-mediated upregulation of vascular endothelial growth factor (VEGF) [82]. Increased ferritin expression in cancer cells is also linked to the acquisition of metastatic and multidrug-resistant phenotypes [83,84].

TNBC cells rely heavily on glutamine, harnessing glutaminolysis to drive biosynthesis, energy production, and antioxidant defense mechanisms, including glutathione (GSH) synthesis [85]. Through overexpression of transporters like ASCT2 (SLC1A5) and LAT1 (SLC7A5), TNBC efficiently imports glutamine, which is then converted by glutaminase (GLS) into glutamate [86,87]. This increase in glutamate production fuels the tricarboxylic acid (TCA) cycle and supports the synthesis of GSH, a key antioxidant that shields cells from oxidative stress. Elevated GLS expression, along with heightened ASCT2 and LAT1 activity, creates a metabolic profile in TNBC marked by low intracellular glutamine and high glutamate, further suggesting enhanced glutaminolysis and dependency on this pathway [88,89]. Another critical protective mechanism in breast cancer cells involves bolstering their antioxidant systems to manage excess iron without succumbing to ferroptosis. Central to this defense is the glutathione (GSH)/glutathione peroxidase (GPX)/glutathione reductase (GR) pathway, which employs the thiol-based antioxidant glutathione (γ-glutamylcysteinylglycine), the selenium-containing enzyme GPX (particularly GPX4), and NADPH as a source of reducing power. TNBC cells increase GSH availability by upregulating the cystine transporter SLC7A11, which facilitates greater cystine uptake and the rate-limiting step for GSH synthesis [90]. Additionally, they stimulate the hexose monophosphate pathway and malic enzyme activity to boost NADPH production, which powers the reduction of GSH [91]. The upregulation of GPX4 further strengthens this system, neutralizing harmful hydroxyl and lipid alkoxyl radicals and thus preventing ferroptotic cell death despite high intracellular iron levels [92].

Building on their role in the tumor microenvironment, tumor-associated macrophages (TAMs) further contribute to ferroptosis resistance in triple-negative breast cancer (TNBC) through a novel pathway involving hepatic leukemia factor (HLF). TAM-derived TGF-β1 regulates HLF, activating gamma-glutamyltransferase 1 (GGT1), a critical enzyme in glutathione metabolism. This pathway boosts the antioxidant defenses of TNBC cells, enabling them to counteract lipid peroxidation and avoid ferroptotic cell death. By facilitating ferroptosis resistance, this mechanism drives TNBC cell proliferation, metastasis, and resistance to cisplatin, contributing to the aggressiveness and therapeutic challenges of these tumors [93].

#### 3.2.2. Achilles’ Heel of Breast Cancer in Ferroptosis

Understanding ferroptosis-based defense mechanisms of breast cancer cells is key to identifying their precise vulnerabilities—Achilles’ heels can be exploited for therapeutic purposes. Moreover, breast cancer cells display strong antioxidant systems and metabolic adaptation, but they contain inbuilt weaknesses to ferroptosis that emerge from metabolic vulnerabilities and imbalances. One of the crucial vulnerabilities is a high polyunsaturated fatty acid (PUFA) level in cell membranes [94]. PUFAs are very labile and especially vulnerable to lipid peroxidation, the beginning of ferroptosis, and their formation into membrane phospholipids is enacted by enzymes such as acyl-CoA synthetase long-chain family member 4 (ACSL4). The activity of ACSL4, therefore, not only influences the structure and dynamics of lipid metabolism in breast cancer cells but also puts them at high risk of triggering ferroptosis. That makes ACSL4 a strategic intervention target [95]. Another critical weakness is the heightened accessibility to labile iron, which has been widely observed in breast cancer cells. Such iron surfeit, a by-product of deranged iron metabolism, fuels the formation of reactive oxygen species (ROS) with the Fenton reaction, thereby amplifying lipid peroxidation and the likelihood of ferroptotic death in the cells [96]. Although iron overload underpins the tumor breath in most cancers, it paradoxically increases susceptibility to ferroptosis. Meanwhile, the glutathione peroxidase 4 (GPX4)–glutathione (GSH) axis, a supreme guardian of ferroptosis, is frequently nonfunctional under oxidative stress and metabolic demands in breast cancer [97,98]. This dysfunctional defense mechanism cannot effectively detoxify lipid peroxides, rendering cancer cells susceptible to oxidative injury and ferroptotic cell death.

Building on earlier discussions of the system Xc- and GPX4 pathways, the next point is to stress metabolic vulnerabilities exacerbated by epithelial–mesenchymal transition (EMT) in aggressive breast cancers, including triple-negative breast cancer (TNBC). EMT reconditions cancer cells, leading them to become so dependent on the cystine–glutamate antiporter system Xc- [99]. This antiporter is essential for importing extracellular cystine, which is critical for glutathione (GSH) synthesis. GSH is critical for reducing lipid peroxides by glutathione peroxidase 4 (GPX4), enabling these cells to evade ferroptosis. The EMT-induced cells have significantly upregulated system Xc- components such as SLC7A11. This scenario reinforces the dependency of EMT-induced cells on cystine that enables them to regulate redox balance under rising oxidative stress conditions [90]. Such an addiction creates a critical vulnerability; pharmacological inhibition of system Xc-, as with erastin, reduces intracellular cystine and GSH levels, thereby inhibiting GPX4 activity. As endogenous cellular defenses fail, the buildup of lipid peroxides accelerates, leading to ferroptotic cell death, which offers a targetable avenue for eliminating mesenchymal-like, therapy-resistant cancer cells [100]. Therefore, exploiting cystine addiction in EMT-induced cells, ferroptosis-inducing therapies prove to be an attractive approach for opposing breast cancer progression and metastasis.

HER2-positive breast cancer cells, much like TNBC, demonstrate distinct susceptibility to ferroptosis, particularly when therapeutic resistance arises. After exposure to lapatinib, a HER2-targeted therapy, certain cells evade initial eradication by adopting a drug-tolerant persister state. These persister cells exhibit an elevated reliance on glutathione peroxidase 4 (GPX4), a crucial enzyme responsible for suppressing lipid peroxidation and preventing ferroptosis. This dependency stems from a compensatory adaptation to their weakened antioxidant capacity and heightened lipid peroxidation. Targeting GPX4 in lapatinib-resistant cells induces selective ferroptotic cell death, effectively eradicating these drug-tolerant populations and diminishing the risk of tumor relapse [101]. These findings highlight GPX4 as a key vulnerability in HER2-positive breast cancer and suggest it as a potential therapeutic target. Integrating ferroptosis-inducing approaches with current HER2-directed therapies could address lapatinib resistance, presenting a novel strategy for managing this aggressive cancer subtype.

### 3.3. Compounds Targeting Ferroptosis in Breast Cancer

#### 3.3.1. Exploring Clinical Potential

The vulnerabilities of breast cancer cells to ferroptosis, as discussed earlier, open intriguing possibilities for therapeutic intervention. Although ferroptosis is not yet a direct focus in clinical oncology, several compounds currently being tested in breast cancer clinical trials may exert effects on the ferroptotic pathway. While not explicitly designed to induce ferroptosis, these agents interact with key regulators such as iron homeostasis, lipid peroxidation, and antioxidant systems. By examining their mechanisms of action, it becomes evident that they could potentially target the ferroptotic vulnerabilities of breast cancer cells. This section explores such compounds, connecting their therapeutic potential to ferroptosis and investigating how they might align with current clinical efforts, thereby bridging preclinical insights with clinical applications.

A novel approach in ferroptosis-based therapy involves using carbon nanoparticle-iron complexes (CNSI-Fe(II)), which exploit the unique iron metabolism of tumor cells to trigger ferroptotic cell death. Free Fe(II) ions face limited intracellular accumulation due to restricted transport via transferrin. Under physiological conditions, iron enters the cytoplasm through transferrin (Tf)-Fe2/transferrin receptor (TFR)-mediated endocytosis or alternative pathways and is subsequently exported by ferroportin (FPN) to maintain systemic homeostasis [102,103]. However, an excess of Fe(II) disrupts this equilibrium, catalyzing the Fenton reaction and generating reactive oxygen species (ROS), which induce oxidative damage [104]. Despite tumors’ reliance on altered iron metabolism for growth, the direct delivery of free Fe(II) is not feasible due to its rapid systemic diffusion and associated toxicity risks [105]. CNSI-Fe(II) overcomes these challenges by adsorbing Fe(II) onto carbon nanoparticles, which tumor cells internalize through endocytosis. Once inside, the aberrant iron-handling mechanisms of cancer cells—characterized by increased Fe(II) uptake and impaired export through the FPN pathway—facilitate iron retention, initiating the Fenton reaction. This localized oxidative stress damages cellular structures, culminating in ferroptotic death [106]. Xie et al. [106] demonstrated in their in vivo study that intratumoral injection of CNSI-Fe significantly inhibited tumor growth in H22 tumor-bearing mice. Tumor volume in the CNSI-Fe-treated group was reduced by approximately 64.7%, surpassing the reduction achieved by cisplatin (DDP), which was 50.3%. Prussian blue staining and elemental analysis confirmed significant Fe deposition within tumor tissues, facilitating the Fenton reaction and generating reactive oxygen species. The resulting ROS induced oxidative stress, extensive necrosis, and structural damage to cancer cells, as demonstrated through histological analysis and transmission electron microscopy (TEM). Notably, CNSI-Fe(II) exhibited minimal systemic toxicity, with Fe accumulation restricted to the tumor site and no notable increase in Fe levels detected in other tissues. These findings underscore the high therapeutic efficacy and low systemic toxicity of CNSI-Fe(II), positioning it as a promising alternative to conventional chemotherapeutics for localized cancer treatment. Phase 1 clinical trial (NCT06048367) is currently underway, employing intratumoral injection of CNSI-Fe(II) complexes to maximize tumor specificity while minimizing systemic exposure and associated risks [107].

In the ongoing search for improved breast cancer treatments, particularly for triple-negative breast cancer (TNBC), combining dasatinib and quercetin with chemotherapy has shown promise in addressing therapy resistance. Dasatinib, a tyrosine kinase inhibitor, disrupts key pathways that drive tumor growth and metastasis [108]. Importantly, it also targets breast cancer stem cells (BCSCs), a subpopulation within tumors that is closely associated with chemotherapy resistance and disease recurrence [109]. Dasatinib enhances the effects of chemotherapeutic agents like paclitaxel by depleting these stem cells. Results from the phase II GEICAM/2010-04 study have demonstrated that this combination not only improves tumor response but also reduces chemoresistance [110]. Additionally, preclinical evidence from ovarian cancer models indicates that dasatinib, when paired with agents like carboplatin or olaparib, effectively reduces metastatic spread to adipose and peritoneal tissues [111]. This highlights its potential relevance to breast cancer treatments, particularly in addressing tumor microenvironment that promotes disease progression. Quercetin, a naturally occurring flavonoid, complements dasatinib by leveraging ferroptosis, an iron-dependent form of cell death. It induces ferritinophagy, a lysosomal process that releases iron ions stored in ferritin, leading to an accumulation of intracellular Fe(II). This excess iron catalyzes the Fenton reaction, producing reactive oxygen species (ROS) that damage cellular membranes through lipid peroxidation and ultimately trigger ferroptosis [112]. The combined effects of dasatinib and quercetin disrupt the redox balance within chemoresistant cancer cells, amplifying their susceptibility to treatment. Phase 2 clinical trial (NCT06355037) is currently investigating this combination, aiming to reverse chemotherapy resistance in TNBC patients [113]. Together, dasatinib and quercetin offer a promising multidimensional strategy for overcoming resistance and improving outcomes in aggressive breast cancer subtypes.

Disulfiram, an FDA-approved drug traditionally used to treat alcoholism, gains anticancer properties when complexed with copper [114,115].] This combination targets triple-negative breast cancer (TNBC) by leveraging oxidative stress mechanisms. It significantly increases the activity of heme oxygenase-1 (HMOX1), an enzyme that catalyzes the degradation of heme, producing ferrous iron. The resulting Fe(II) ions, especially in the mitochondria, enhance the Fenton reaction that produces ROS, triggering lipid peroxidation and leading to mitochondrial damage and ferroptosis in cancer cells [116]. At the same time, disulfiram-copper treatment reduces the number of key antioxidant molecules, such as glutathione (GSH) and glutathione peroxidase 4 (GPX4), that fight ferroptosis on the cellular level [117]. Preclinical studies support its efficacy in inducing ferroptotic cell death in TNBC models, and a phase 2 clinical trial (NCT03323346) is currently underway to assess its therapeutic potential in metastatic breast cancer [118].

Sulfasalazine (SSZ), an orally administered anti-inflammatory medication commonly prescribed for the treatment of inflammatory bowel disease and rheumatoid arthritis, is another promising intervention in ferroptosis-oriented therapy in breast cancer [119,120]. Apart from its anti-inflammatory properties, it is an Xc- system inhibitor, which inhibits the exchange of extracellular cystine for intracellular glutamate that has a crucial role in glutathione (GSH) synthesis [121]. This disruption leads to reduced intracellular GSH levels, compromising the glutathione peroxidase 4 (GPX4)-mediated defense against lipid peroxidation, thus inducing ferroptosis. In addition to its ferroptotic effects, sulfasalazine diminishes glutamate release by breast cancer cells, which is particularly significant for managing cancer-induced bone pain (CIBP) [122,123]. Excess glutamate outside of cells is recognized as a signaling molecule, and its heightened levels can activate NMDA receptors and sensitize nociceptive pathways, leading to chronic pain in individuals with bone metastases [124]. Ungard et al. [125] discovered that SSZ effectively reduced glutamate release from MDA-MB-231 breast cancer cells in vitro. In vivo, studies demonstrated that sulfasalazine not only mitigates nociceptive behaviors in animal models of breast cancer bone metastases but also delays the onset of pain symptoms [125]. These findings underscore the dual benefits of sulfasalazine, targeting both cancer cell survival through ferroptosis induction and improving patient quality of life by alleviating cancer-associated pain. Phase 2 clinical trial (NCT03847311) evaluated sulfasalazine’s efficacy in reducing cancer cell viability and its potential to decrease opioid dependency by controlling CIBP in breast cancer patients [126].

Simvastatin and fluvastatin, commonly prescribed statins for hypercholesterolemia, are being explored for their potential anticancer properties, particularly in breast cancer [127,128]. Statins function by inhibiting 3-hydroxy-3-methylglutaryl-coenzyme A reductase (HMGCR), a key enzyme in the mevalonate (MVA) pathway [129]. This pathway regulates the synthesis of isoprenoids like isopentenyl pyrophosphate (IPP), crucial for the post-translational modification of selenoproteins, including glutathione peroxidase 4 (GPX4) [130]. GPX4 is essential for mitigating oxidative stress by detoxifying lipid peroxides, thereby preventing ferroptosis. By blocking HMGCR activity, simvastatin disrupts the MVA pathway, leading to impaired IPP production and inadequate GPX4 function, potentially sensitizing cancer cells to ferroptosis [96]. Statins preferentially target tumor cells with mesenchymal-like characteristics that arise through epithelial-to-mesenchymal transition (EMT) [131]. This process enhances cellular motility and metastatic capabilities, simultaneously creating metabolic dependencies that render these cells vulnerable to MVA pathway inhibition. By inhibiting the mevalonate pathway, statins disrupt protein N-glycosylation, a critical process for proper cell signaling and survival [132]. This disruption compromises glycan branching in the Golgi apparatus and reduces the functionality of cell surface receptors, weakening key pathways that drive metastasis. Yu et al. have demonstrated the effectiveness of this approach; in a mouse model of postsurgical metastatic breast cancer, fluvastatin significantly delayed metastatic outgrowth and reduced lung tumor burden, resulting in a survival improvement of over 30% [133]. Given their established safety profile and affordability, statins represent a compelling option for clinical use. Ongoing research, including a Phase 2 trial (NCT05550415) on simvastatin in breast cancer, aims to build on these findings, potentially positioning statins as valuable tools to combat metastatic progression and improve outcomes for patients with aggressive breast cancer subtypes [134].

Metformin, a cornerstone of diabetes management, has shown intriguing potential in breast cancer therapy, also through its ability to induce ferroptosis [135]. By inhibiting mitochondrial complex I, metformin disrupts cellular energy production and places cancer cells in a metabolically stressed state, making them more vulnerable to oxidative damage [136]. Furthermore, metformin affects the cystine-glutamate antiporter system Xc- by inhibiting its methylation and reducing its transport activity. The resulting glutathione depletion weakens antioxidant defenses, allowing ROS and Fe(II) ions to accumulate, triggering lipid peroxidation and ferroptotic cell death. When combined with sulfasalazine, an already discussed system Xc- inhibitor, metformin demonstrates synergistic effects in targeting both metabolic and redox imbalances, as evidenced in preclinical in vivo models [137]. Metformin’s anticancer potential extends to HER2-positive breast cancer, as highlighted by the ALTTO Phase III trial. In this study, metformin inhibited HER2 signaling and reduced cancer stem cell populations, addressing two critical drivers of therapy resistance and metastasis [138]. These findings suggest a possible next step: integrating sulfasalazine into metformin-based therapies to amplify ferroptosis induction and improve treatment outcomes. This dual-action strategy positions metformin as a promising candidate for combination therapies targeting aggressive breast cancer subtypes.

Deferoxamine (DFO), an iron-chelating agent, has attracted significant interest for its potential applications in breast cancer treatment [139]. Cancer cells rely heavily on iron to fuel their rapid growth and metabolic demands, making them particularly vulnerable to disruptions in iron homeostasis. By binding intracellular iron, DFO interferes with critical processes such as DNA synthesis, increasing oxidative stress and triggering apoptosis [140,141]. Intriguingly, DFO has also been found to inhibit ferroptosis, a form of cell death driven by iron-dependent oxidative damage. This effect is achieved by reducing the bioavailability of iron, which subsequently upregulates the system Xc-/GPX4 axis, protecting cells from glutathione (GSH) depletion and preventing the buildup of lipid reactive oxygen species [142,143]. Beyond its direct effects on cell viability, DFO’s ability to lower intracellular iron levels may also enhance the sensitivity of breast cancer cells to chemotherapy, creating new opportunities for therapeutic intervention [144]. The ongoing phase 2 clinical trial (NCT05300958) is now exploring the role of DFO in breast cancer, aiming to understand better its potential to improve treatment outcomes [145].

#### 3.3.2. Insights from In Vivo Studies

After investigating the advancements in clinical trials and preclinical research, the next logical progression is to evaluate drugs tested in vivo that demonstrate the potential to induce ferroptosis. This shift from laboratory discoveries to applied research offers valuable insights into how existing therapies can be optimized or combined to improve treatment outcomes in breast cancer. One of the notable examples is doxorubicin (DOX). This commonly used chemotherapy drug has been shown to trigger ferroptosis in triple-negative breast cancer (TNBC) models when used in conjunction with specific metabolic inhibitors. This section will explore the mechanisms and findings of in vivo studies, starting with the intriguing interaction between doxorubicin and ferroptosis pathways. Chemotherapy-resistant TNBC cells, which rely heavily on glutaminolysis, demonstrate a notable capacity to sustain low levels of cellular superoxide and lipid peroxidation during oxidative stress [146]. This resilience is linked to their adaptive metabolic rewiring. However, a dual metabolic inhibition approach—targeting glutaminase with CB839 and the cystine/glutamate antiporter system Xc- with erastin—disrupts this balance by depleting glutathione and increasing ROS accumulation. When paired with DOX, this approach significantly enhances ferroptotic cell death, as shown by an 80% drop in glutathione levels, elevated lipid peroxidation, and substantial tumor growth suppression in vivo [147]. Additionally, DOX independently augments ferroptosis by facilitating iron uptake and synthesis while reducing the activity of iron efflux transporters, thereby exacerbating oxidative damage [148]. These findings highlight the potential of combining DOX with metabolic inhibitors to overcome resistance and target vulnerabilities in TNBC cells.

Neratinib, a potent irreversible tyrosine kinase inhibitor (TKI), has become a noteworthy therapeutic option for HER2-positive breast cancer [149,150]. Approved in 2020 based on findings from the Phase III NALA clinical trial, neratinib was shown to significantly extend progression-free survival (PFS) and decrease the incidence of central nervous system (CNS) metastases in patients previously treated with two or more HER2-directed therapies [151,152]. By irreversibly inhibiting HER family receptors, including HER2 and EGFR, neratinib ensures prolonged suppression of signaling pathways that drive cancer growth and survival [153]. In a syngeneic mouse model (TBCP-1), neratinib exhibited strong and sustained HER2 inhibition, resulting in ferroptosis characterized by iron accumulation and lipid peroxidation. This form of cell death was validated by the protective action of liproxstatin-1, a known ferroptosis inhibitor, which mitigated neratinib-induced cytotoxic effects. Further investigations demonstrated that neratinib disrupts iron homeostasis, as reflected by changes in key iron regulators such as ferritin and ferroportin-1. Importantly, this pro-ferroptotic activity appears to be specific to neratinib compared to other tyrosine kinase inhibitors, underscoring its unique mechanism of action. Although the exact pathways remain to be fully understood, these results indicate that neratinib’s dual function as a HER2 inhibitor and ferroptosis inducer offers promising potential for overcoming treatment resistance and addressing metastatic HER2-positive breast cancer [154].

Traditionally used for its anti-inflammatory and analgesic effects in treating venous circulation disorders and post-traumatic injuries, escin has recently emerged as a potential anticancer agent [155,156,157]. Li et al. [158] showed that escin can induce ferroptosis by reducing the GSH (reduced glutathione)/GSSG (oxidized glutathione) ratio and increasing ROS and lipid peroxidation levels in vitro. It also facilitated the ubiquitination of glucose-6-phosphate dehydrogenase (G6PD), a critical enzyme of the pentose phosphate pathway, contributing to the repression of GPX4, a significant antioxidant against ferroptosis [159]. Additionally, escin was dose-dependently synergic with cisplatin, improving the effectiveness of this common chemotherapeutic drug to kill tumor cells. G6PD’s role in the antitumor effect of escin was evaluated in a xenograft model using MDA-MB-231 cells with G6PD knockdown or control vectors. Mice treated with escin or G6PD knockdown alone showed mild tumor growth inhibition, but the combination of G6PD knockdown and escin resulted in significantly enhanced tumor suppression. Reduced expression of G6PD, GPX4, and Ki67 in xenografts further validated the antitumor effects, demonstrating that G6PD inhibition amplifies escin’s efficacy in vivo [158].

A promising minimally invasive treatment for breast cancer is photodynamic therapy (PDT); however, its effectiveness is limited by the tumor microenvironment (TME), which is characterized by hypoxia and elevated glutathione (GSH) levels. In the study demonstrated by Yi Sun et al. [160] PDT was enhanced on 4T1 cell line (TNBC in vitro and in vivo model) by adding K_2_FeO_4_ before the irradiation. This approach induced breast cancer killing via in situ synthesis of Fe_2_O_3_ and O_2_ within the tumor. Ce6-mediated PDT was enhanced through synergistic ferroptosis. By promoting ROS generation, inducing lipid peroxidation, and suppressing GSH and GPX4, this method significantly improves PDT in vitro efficacy [160].

#### 3.3.3. Insights from In Vitro Studies

Lastly, we will delve into in vitro studies focusing on commonly used drugs and photosensitizers that may induce ferroptosis but require further evaluation through in vivo investigations. These studies offer fundamental insights into the mechanisms of action and potential therapeutic applications, setting the stage for future research to validate their therapeutic effectiveness in more complex biological systems.

Lapatinib, a tyrosine kinase inhibitor, has become a vital treatment for HER2-positive breast cancer, particularly in patients who no longer respond to trastuzumab [161]. Beyond its established role in targeting HER2 signaling, recent findings suggest that lapatinib can induce ferroptosis, especially when combined with siramesine. Siramesine, a lysosomal disruptor, works in tandem with lapatinib to increase intracellular iron levels and amplify reactive oxygen species (ROS), creating an environment of heightened oxidative stress. Together, these effects disrupt iron homeostasis and promote lipid peroxidation, key markers of ferroptosis. Lapatinib contributes by upregulating transferrin receptors, which enhance iron uptake, while siramesine destabilizes lysosomes and induces ferritin degradation via autophagy [162,163]. This dual action not only intensifies ferroptosis but also targets explicitly therapy-resistant cancer cells, offering a promising strategy to address the challenges of HER2-positive breast cancer.

Propofol, commonly used as an intravenous anesthetic for its sedative and pain-relieving characteristics during surgery, has been found in recent research to cause ferroptosis in MDA-MB-231 cells. Sun et al. [164] found that propofol, both alone and in combination with chemotherapeutics like doxorubicin and paclitaxel, enhanced cell death and significantly suppressed proliferation. The combination therapies exhibited a synergistic effect, with propofol amplifying chemotherapeutic sensitivity, promoting ROS accumulation, lipid peroxidation, and elevated intracellular iron accumulation. Furthermore, transmission electron microscopy confirmed ferroptotic mitochondrial shrinkage, supporting the hypothesis that propofol augments ferroptosis to inhibit cancer cell growth [164]. However, further research is needed to thoroughly understand the molecular pathways it targets and confirm its effectiveness in vivo. These findings highlight an exciting opportunity to repurpose propofol as a potential tool to enhance the impact of existing cancer therapies.

In Table 1 we present the summary of compounds targeting ferroptosis in breast cancer. It is important to acknowledge that many of the compounds summarized in Table 1 exert biological effects beyond ferroptosis. Agents such as metformin, statins, and doxorubicin have well-established roles in modulating apoptosis, autophagy, and metabolic stress responses. Their ability to induce ferroptosis, while promising, therefore represents only one dimension of their broader anticancer activity. Consequently, attributing their therapeutic efficacy solely to ferroptosis would oversimplify their mechanisms of action.

Moreover, ferroptosis does not occur in isolation within the tumor microenvironment. Other regulated forms of cell death—including apoptosis, necroptosis, and autophagy—may act in parallel or interact with ferroptotic pathways. For example, oxidative stress and mitochondrial dysfunction can trigger both apoptotic and ferroptotic cascades, while autophagy contributes to ferroptosis by regulating ferritin degradation (ferritinophagy) and iron release. Conversely, in some contexts, autophagy may serve a cytoprotective role by limiting lipid peroxidation. Given the complexity of breast tumors, especially under hypoxic and nutrient-limited conditions, it is likely that multiple cell death programs contribute to treatment responses. This underscores the need for careful mechanistic studies to disentangle ferroptosis-specific effects from broader cell death responses.

### 3.4. Discussion

The literature research reveals that inducing ferroptosis is a promising therapeutic strategy for the treatment of aggressive malignancies, especially triple-negative breast cancer (TNBC) and HER2-positive breast cancer. Moreover, studies show that the tumor microenvironment conditions and mechanisms of chemotherapy resistance may also be overcome by ferroptosis induction. The most important advantage of this cell death activation is its selective disruption of targeted cells even in hypoxic areas with minimal effect on healthy tissues. The main characteristics of breast cancer cells that can undergo ferroptosis include a strong dependency on the cystine–glutathione–GPX4 axis as a defense against lipid peroxidation, high levels of polyunsaturated fatty acids (PUFAs) in the cell membranes, and an abundance of iron, which are among the main weaknesses of breast cancer cells. Nevertheless, several aggressive tumors exhibit similar ferroptosis mechanisms to those seen in breast cancer. Based on this knowledge, it is of utmost importance to translate these findings from one malignancy to another.

For the first time, the process of ferroptosis was described by Dixon SJ et al. [21], who presented foundational work on erastin—an inhibitor of GPX4 that induces ferroptosis in HT-1080 cells, a highly aggressive fibrosarcoma cell line. Following their research, other malignant cells were also evaluated for susceptibility to iron-dependent cell death. One of the most important organs in iron accumulation is the liver. Therefore, hepatocellular carcinoma (HCC) cells provide an excellent model to study iron-dependent cell death due to their extreme sensitivity to cysteine transport blockade. Studies conducted both in vitro and in vivo on HCC cells have demonstrated that the combination of chemotherapy with GPX4 inhibitors inhibits tumor growth and increases cell death. Agents such as RSL3 or the blockade of cystine uptake by erastin or sulfasalazine can effectively trigger ferroptotic cell death in HCC tumors [165]. Therefore, strategies presented in the review that use combination treatments to induce ferroptosis in breast cancer cells validate this promising approach for future studies.

A further widely explored cancer model for studying ferroptosis as a therapeutic target is pancreatic ductal adenocarcinoma (PDAC). PDAC cells exhibit extremely strong antioxidant defenses and enhanced glutaminolysis; however, inhibition of cysteine uptake has been shown to still usefully trigger ferroptosis and suppress tumor growth [166].

Additionally, several compounds have been reported to induce ferroptosis in PDAC cells, including artesunate, cyst(e)inase, EGCG, imidazole ketone erastin, piperlongumine, and the antiviral drug zalcitabine, which differ from the ferroptosis inducers typically studied in breast cancer [167]. Repurposing these agents for breast cancer therapy may represent an appealing strategy to eliminate tumor cells via ferroptotic pathways.

On the other hand, erastin and sulfasalazine—agents that inhibit cystine transport, reduce GSH levels, and thereby promote ferroptosis—have already shown promising effects in TNBC and HER2-positive breast cancer models. Nevertheless, research by Li et al. demonstrated that erastin also induces clusterin overexpression in PDAC, which in turn significantly promotes cell proliferation and acts as a cytoprotective factor [168]. This suggests that the same drugs may have variable effectiveness in ferroptosis induction across different cancer types. This PDAC example highlights that ferroptosis inducers may paradoxically activate survival pathways, thus indicate the need for further investigation in TNBC model. This variability among breast histological types pointed out the necessity of identifying for example predictive biomarkers for patient stratification to evaluate who are most likely to benefit from ferroptosis-targeted therapies.

Based on the research by Hangauer et al. [101], inhibition of GPX4 increases lipid peroxidation, which in turn activates ferroptosis in the HER2-positive breast cancer cell line BT474. Other studies have shown that, due to their reliance on GPX4, glioma stem cells are also highly vulnerable to ferroptosis. Minami et al. [169] demonstrated, in both in vitro and orthotopic xenograft models of patient-derived glioblastoma (GBM), that ferroptosis may represent a promising therapeutic target in this tumor type. The key mechanism involves deletion of the CDKN2A gene, which alters the protective role of polyunsaturated fatty acids (PUFAs) in buffering oxidative stress. Their work indicates that loss of CDKN2A sensitizes GBM cells to ferroptotic cell death. On the other hand, the same study by Hangauer et al. reveals that in ER-positive breast cancer models such as MCF-7, there is low cell response due to differences in SLC7A11 expression, lipid composition, and antioxidant capacity.

Another important study by Li S. et al. [170] further explored ferroptosis in GBM by targeting the glutathione pathway. Using the well-known GPX4 inhibitor RAS-selective lethal 3 (RSL3), they revealed and confirmed a novel therapeutic strategy for glioblastoma based on activation of the NF-κB pathway, which plays a previously unrecognized role in RSL3-induced ferroptosis.

Furthermore, induction of ferroptosis represents an effective strategy to overcome the problem of chemotherapy resistance. For instance, Xiumei Chen et al. [171] demonstrated that docetaxel-resistant prostate cancer cell lines (PC3-DR, DU145-DR, and VCaP-DR) treated with ferroptosis inducers—erastin and RSL-3—exhibited a greatly enhanced cytotoxic response to chemotherapy. Taken together, this finding provides strong evidence that conjugation of chemotherapeutic agents with ferroptosis inducers is a promising strategy not only for resistant breast cancers but also for other malignancies.

The aforementioned examples indicate that ferroptotic pathways, including GPX4 dependence, the Xc-/GPX4 antioxidant system, and the regulation of intracellular iron, are comparable in breast cancer and other aggressive cancer types. This highlights the translational potential of ferroptosis research, as therapeutic approaches developed for one tumor type may be adapted for use in others.

It should be noted that many substances listed in Table 1 may have biological effects that are not related to ferroptosis. For instance, it is known that plant compounds like artesunate, curcumin, or piperlongumine can alter mitochondrial activity, oxidative stress, or signaling cascades other than ferroptotic pathways. Furthermore, in the tumor microenvironment, other death pathways such as necrosis, autophagy, and apoptosis may be involved. This demonstrates that the drug action may be pleiotropic and may contribute to the effectiveness of the treatment of breast cancer models [172,173]. Therefore, future research should concentrate on separating these overlapping pathways in order to elucidate the precise role that ferroptosis plays in tumor suppression.

Taken together, these contradictory and context-dependent findings highlight both the promise and complexity of targeting ferroptosis in breast cancer. Insights from other cancers, while valuable, must be interpreted with caution when applied to breast cancer. Hepatocellular carcinoma, for instance, is more vulnerable to ferroptosis because the liver is a major site of iron storage and metabolism. Similarly, glioblastoma (GBM) studies have limited potential by the uniqnes of brain tumors and the brain–blood barrier. These examples emphasize that direct extrapolation is limited. A more nuanced understanding of compensatory pathways (e.g., clusterin signaling), subtype-specific vulnerabilities, genetic and tumor microenvironment interactions will be essential to translate ferroptosis-based approaches into clinically meaningful strategies.

There are some technical limitations of the presented review. Most of the available data are derived from in vitro experiments using a limited number of breast cancer cell lines (e.g., MCF-7, 4T1, BT474), which raises concerns about selection bias and limits the generalizability of the findings. Performance bias may also be present, as experimental protocols differ considerably regarding ferroptosis inducers, concentrations, and time of exposure. Furthermore, detection bias cannot be excluded, given the heterogeneity of methods applied to assess ferroptosis (ROS accumulation assays, lipid peroxidation markers, GPX4 expression).

As discussed in this review, patients with TNBC and HER2-positive breast cancer may in the future benefit from ferroptosis induction combined with traditional chemotherapy, natural compounds, immunotherapy, photodynamic therapy or nanoparticle-based strategies. Enhanced treatment efficacy, the ability to overcome drug resistance, and the potential to reduce metastasis may contribute to defeating these fast-growing and highly aggressive breast cancer subtypes.

## 4. Conclusions

In conclusion, research on ferroptosis in breast cancer is strongly supported by data from other malignancies. Induction of ferroptosis represents a promising avenue for selective therapy, particularly in tumors resistant to apoptosis-based treatments. Nevertheless, further clinical studies are required to determine the most effective strategies, especially for triple-negative breast cancer (TNBC) and HER2-positive breast cancer.

## Figures and Tables

**Table 1:** Compounds targeting ferroptosis in breast cancer.

Drug	Mechnism of Action	Study Type	Reference
CNSI-Fe(II)	Increases intracellular iron levels, triggering the Fenton reaction leading to ROS production.	Clinical trial	Clinical trial NCT06048367 [107]
Dasatinib + Quercetin	Targets breast cancer stem cells; induces ferritinophagy, increasing intracellular iron levels. intracellular iron levels.	Clinical trial	Clinical trial NCT06355037 [113]
Disulfiram + Copper	Enhances heme degradation, increasing intracellular iron levels; reduces GSH and GPX4 levels.	Clinical trial	Clinical trial NCT03323346 [118]
Sulfasalazine	Inhibits system Xc-, reducing intracellular GSH levels.	Clinical trial	Clinical trial NCT03847311 [126]
Simvastatin/Fluvastatin	Inhibits the mevalonate pathway, impairing GPX4 function.	Clinical trial	Clinical trial NCT05550415 [134]
Metformin	Disrupts mitochondrial function and inhibits system Xc-.	Clinical trial	Sonnenblick et al. [138]
Doxorubicin + CB839 + Erastin	Inhibits glutaminase and system Xc-, reducing intracellular GSH levels	In vivo	Choi et al. [147]
Neratinib	Disrupts iron homeostasis, leading to iron accumulation and lipid peroxidation.	In vivo	Nagpal et al. [154]
Escin	Reduces GSH/GSSG ratio and increases ROS, leading to lipid peroxidation; inhibits the pentose phosphate pathway impairing GPX4 function.	In vivo	Li et al. [158]
K_2_FeO_4_ following Ce6-mediated PDT	Promotes ROS generation, induces lipid peroxidation and suppresses GSH and GPX4.	In vivo and in vitro	Sun et al. [160]
Lapatinib + Siramesine	Increases intracellular iron levels, promoting ROS accumulation and lipid peroxidation.	In vitro	Ma et al. [162] Ma et al. [163]
Propofol	Increases intracellular iron levels, promoting ROS accumulation and lipid peroxidation	In vitro	Sun et al. [164]

## Data Availability

No new data were created or analyzed in this study. Data sharing is not applicable to this article.
